# The use of multivariate PCA dataset in identifying the underlying drivers of critical stressors, looking at global problems through a local lens

**DOI:** 10.1016/j.dib.2022.107946

**Published:** 2022-02-11

**Authors:** Junghyung Ryu, Kam-biu Liu, Terrence A. McCloskey

**Affiliations:** a Department of Oceanography and Coastal Sciences, College of the Coast and Environment, Louisiana State University, Energy, Coast & Environment Building, Baton Rouge, LA 70803, USA; b St. Margarets Village, Mile 32 Hummingbird Highway, Belize, USA

**Keywords:** Palynology, Geochemistry, Multivariate principal component analysis, Coastal science, X-ray fluorescence

## Abstract

Palynology-based multivariate datasets including geological, ecological, and geochemical data identified the relative importance of the underlying drivers of critical stressors to coastal wetlands by identifying and distinguishing between fluvial flooding, saline water intrusion, delta switching, and the landward migration of coastal plants. A sediment core was retrieved using a vibracorer from an intermediate marsh in Lake Salvador, Louisiana, USA. X-ray Fluorescence (XRF) quantified fluvial and marine elemental concentrations (Cl, Sr, Ca, Mn, K, Ti, Fe, Zn, Zr, Br). Palynology-based agglomerate hierarchical analysis of thirty-two pollen taxa was employed to define ecological clusters. The implementation of multivariate principal component analysis (PCA) to geochemical and ecological variables inferred the source of sedimentary material by correlating four taxonomic groups (floodplain trees, upland trees, tidal freshwater herbs, and inland herbs) to specific geochemical signatures and facilitated the testing of potential correlations between geo- and hydrological-conditions and the six ecosystems (interdistributary, delta-plain, deltaic lake, bottomland and swamp forests, freshwater marsh, and intermediate marsh) depicted in each PCA biplot. The PCA scores quantified the relative importance of multiple variables. The squared cosine function, which demonstrates the relative importance of a variable for a given observation, was used to estimate the representation of each variable on the principal component biplots. Multivariate statistical datasets can be valuable to any scientist working across the spectrum of environmental and planetary science fields as a means of identifying the relative importance of diverse background parameters in controlling ecological and environmental conditions. This methodology is applicable across both natural and social sciences as a means of distinguishing natural and anthropogenic impacts.

## Specifications Table

**Table:** 

Subject	Earth and Planetary Science
Specific subject area	Multivariate PCA data set identifying mechanisms (e.g., subsidence, eustatic sea-level rise, tropical cyclones) driving coastal environmental changes
Type of data	Table Figure
How the data were acquired	*Sediment coring*: A 448 cm long sediment core (LSWMA) was collected using a vibracorer. *Geochemistry*: X-ray Fluorescence (XRF): An Innov-X Delta Premium DP-4000 X-ray fluorescence analyzer (XRF) was used to scan the core. *Palynological analysis*: Ninety-one samples (1.8 cc each) collected at 5-cm intervals were analyzed using a light microscope. The chemical treatment involved the use of 10% hydrochloric acid (HCl), 10% potassium hydroxide (KOH), 49% hydrofluoric acid (HF), acetolysis solution, glacial acetic acid, and tertiary butanol alcohol (TBA) (Faegri and Iversen, 1975).
Data format	Raw
Description of data collection	*Sediment coring*: The core (LSWMA) was retrieved from an intermediate marsh, surrounded by spikerush (*Eleocharis macrostachya*), broadleaf maidencane (*Panicum hemitomon*), alligator weed (*Alternanthera philoxeroides*), arrowhead (*Sagittaria latifolia*), and cattail (*Typha*). *Geochemistry*: The XRF analyzer utilizes spectral high-resolution in the 8–60 keV region and measures concentrations in parts per million (ppm) of 32 elements. Calibration was performed by scanning National Institute of Standards and Technology (NIST) standards 2710a and 2711a. Soil-Br Mode was operated with the light element analysis program. Scans were 90 s in duration and at a 2-cm resolution down the core. Highly skewed distributions were minimized using log transformation. *Palynological analysis*: A minimum of 300 pollen grains were counted for each sample using a light microscope and charcoal fragments > 10 µm were counted. Published guides (e.g., [1], [2], [3], [4], [5],) were used to aid pollen identifications. *Multivariate Principal Component Analysis (PCA)*: Multivariate PCA was performed using software R packages (e.g., “rioja”, “factoextra”, “FactoMineR”) [6] and a constrained single-link cluster analysis was used to distinguish ecological cluster [7,8], which groups objects (pollen species) in clusters based on their similarity.
Data source location	*Institution: Louisiana State University* *City/Town/Region: Baton Rouge, Louisiana* *Country: United States* *Latitude and longitude for the collected samples/data: 29°46′15.37″N, 90°15′4.11″W*
Data accessibility	Repository name: *Mendeley Data* Data identification number: DOI: 10.17632/m24k64kpw8.1 Direct URL to data: https://data.mendeley.com/datasets/m24k64kpw8/1
Related research article	J. Ryu, K. Liu, T.A. Bianchette, T. McCloskey, Identifying forcing agents of environmental change and ecological response on the Mississippi River Delta, Southeastern Louisiana, Science of The Total Environment 794 (2021) 148,730. https://doi.org/10.1016/j.scitotenv.2021.148730.

## Value of the Data

• Multivariate (ecological, geological, and chemical) statistical data, designed for coastal and environmental studies, provide valuable information to distinguish the relative importance of background parameters in controlling the environmental conditions, which was then used to infer the responsible external forcing agents.
• Multivariate statistical datasets can be useful to any scientist working in environmental and planetary science fields as a means of identifying the relative importance of background parameters in controlling ecological and environmental conditions. This methodology is applicable across both natural and social sciences as a means of distinguishing natural and anthropogenic impacts.
• The datasets can be used in all scientific fields dealing with such pressing global problems as oceanic transgression, species extinction, global warming, the abundance of plastic trash, etc. It provides a starting point and points of attack for devising solutions. This approach can infer hidden forcing agent(s) if the relevant dataset provides sufficient temporal and/or spatial coverage.

## 1 Data Description

**Fig. 1:**
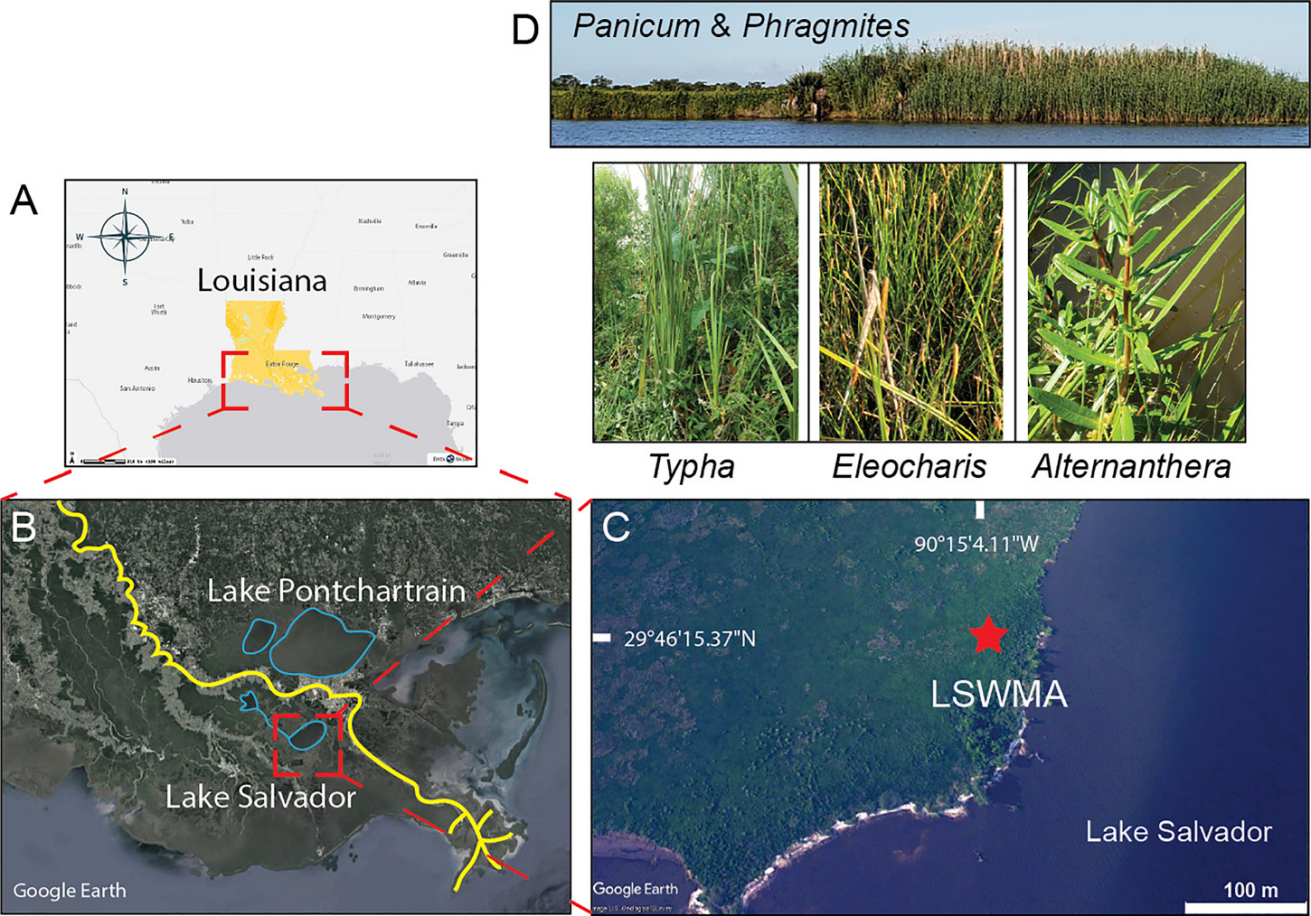
Maps of study site and plants. (A) the state of Louisiana is marked in yellow. (B) satellite image shows the study region and lakes (Lake Salvador and Lake Pontchartrain) marked with blue circles. A yellow line marks the Mississippi River. (C) satellite image displays the sampling site (red star: LSWMA) on the margin of Lake Salvador. (D) photographs of important plant taxa taken in the field. Images were downloaded from data basin (A) and Google Earth (B, C).

This data collection presents a total of 2821 entries of fossil pollen dataset (Table 1), 328 ecological (pollen sums), 820 geochemical (marine and terrestrial elements) datasets (Table 2), and six ecosystem categories (Table 3). All ecological and geochemical datasets are retrieved from the LSWMA core, which was collected from an intermediate marsh encompassing a variety of plant types, including broadleaf maidencane (*Panicum hemitomon*), spikerush (*Eleocharis macrostachya*), alligator weed (*Alternanthera philoxeroides*), cattail (*Typha*), and arrowhead (*Sagittaria latifolia*). The map of the study site (Fig. 1) shows the coring location and plant types. Table 1 provides the fossil pollen dataset in percentages based on palynological analysis of ninety-one samples. Pollen taxa (> 2%) include arboreal taxa (*Pinus, Quercus, Salix, Carya*, Betulaceae, *Myrica, Populus, Nyssa, Taxodium, Fraxinus, Platanus, Liquidambar*, Rosaceae, *Ulmus, Morus, Cephalanthus*, Vitaceae, Caprifoliaceae), and non-arboreal taxa (Cyperaceae, Poaceae, Amaranthaceae, *Ambrosia*, Asteraceae, *Artemisia, Ipomoea*, Fabaceae, Polygonaceae, Nymphaeceae, *Sagittaria, Eichhornia, Typha*, and Araceae).

**Table 1:** Thirty-two pollen taxa.

**Table 2:** Ecological and geochemical data set.

	Ecological data frame					Geochemical data frame									
Number	Floodplain trees	Upland trees	Tidal freshwater herbs	Insland herbs	Number	Cl	Sr	Ca	Mn	K	Ti	Fe	Zn	Zr	Br
1	16.611296	1.993355	44.186047	34.551495	1	739	40	2473	304	2304	579	7515	46	65	358
2	5.128205	8.653846	30.769231	50.320513	2	801	31	2254	1206	1805	439	7511	21	35	315
3	15.159574	6.117021	17.021277	56.914894	3	854	55	1856	265	1931	442	5568	23	75	243
4	11.979167	6.250000	25.000000	54.687500	4	481	29	324	154	753	145	3096	12	37	154
5	10.869565	5.978261	30.163043	51.358696	5	550	56	1109	119	1712	430	3622	13	82	188
6	6.770833	7.031250	23.177083	37.239583	6	414	56	1574	79	2434	690	5700	13	82	97
7	16.216216	19.819820	18.018018	43.243243	7	375	134	3221	103	5082	866	4700	11	200	27
8	11.070111	5.535055	15.498155	59.409594	8	290	76	1168	114	4648	1455	12,711	34	149	23
9	10.032362	2.912621	14.239482	65.372168	9	419	66	819	102	3569	1243	11,000	32	129	22
10	12.244898	1.457726	16.909621	60.058309	10	474	86	1682	146	6276	1752	13,891	31	166	26
11	11.748634	1.366120	13.661202	58.743169	11	458	70	1422	129	5434	1554	13,684	40	132	36
12	12.537313	1.492537	14.925373	56.417910	12	369	77	1415	123	5704	1576	14,264	59	142	41
13	11.838006	1.246106	16.822430	59.190031	13	483	73	1533	124	5928	1622	15,431	59	139	31
14	11.370262	3.790087	19.533528	57.434402	14	518	65	1268	116	4470	1359	13,176	30	130	21
15	11.748634	4.371585	24.863388	53.825137	15	516	75	1705	136	6578	1741	13,837	43	138	48
16	8.978328	6.191950	17.956656	55.417957	16	596	74	1032	121	4077	1289	13,032	36	134	52
17	12.209302	9.302326	16.279070	53.488372	17	383	76	1400	122	4735	1347	12,985	43	133	52
18	14.713896	12.261580	14.713896	50.681199	18	472	54	1005	114	3589	986	11,211	24	105	44
19	6.764706	18.529412	15.000000	53.529412	19	401	68	1467	132	4822	1277	11,981	35	114	70
20	13.333333	11.515152	15.151515	53.636364	20	529	60	589	107	2962	897	10,149	25	104	51
21	13.311688	8.116883	12.337662	57.467532	21	524	67	675	101	3161	1011	10,555	39	122	87
22	15.605096	6.369427	11.783439	56.687898	22	687	66	652	115	2571	846	11,952	65	113	92
23	12.693498	4.643963	15.170279	59.133127	23	351	59	1406	143	4058	972	12,212	27	96	233
24	10.835913	4.334365	18.885449	59.442724	24	280	62	1292	132	5442	1292	12,478	32	102	104
25	14.454277	4.719764	13.569322	61.356932	25	216	61	665	118	3287	1055	12,152	33	112	95
26	16.022099	18.784530	9.944751	51.104972	26	293	52	948	123	2737	762	9209	26	82	165
27	17.079890	25.619835	9.917355	43.250689	27	384	56	901	114	3412	982	10,981	33	93	118
28	22.955145	17.678100	8.179420	48.812665	28	559	66	933	105	3543	913	9903	21	116	109
29	24.739583	15.104167	7.552083	49.739583	29	308	62	835	117	4080	1104	10,637	28	105	108
30	26.388889	16.666667	7.777778	46.666667	30	262	52	747	118	3280	944	11,861	25	93	122
31	31.125828	20.860927	8.609272	37.417219	31	327	48	1610	143	2797	767	11,170	28	64	173
32	24.793388	16.528926	9.641873	48.209366	32	261	52	961	141	3239	894	11,008	29	88	122
33	27.560976	10.487805	10.000000	50.731707	33	270	61	726	110	3892	1126	12,445	38	102	84
34	27.745665	9.537572	8.381503	52.312139	34	283	66	785	113	4510	1244	11,697	30	121	90
35	15.131579	12.171053	17.763158	52.960526	35	506	49	508	117	2214	685	9709	33	81	136
36	27.826087	19.130435	18.550725	34.202899	36	255	57	636	134	3001	999	12,574	34	102	93
37	33.606557	10.109290	9.289617	45.355191	37	316	74	1000	125	4980	1362	12,938	43	142	42
38	24.011299	25.706215	22.033898	24.011299	38	300	47	844	118	3040	922	10,735	28	82	89
39	28.654971	16.666667	9.064327	42.397661	39	311	48	564	114	3433	1008	10,921	33	83	53
40	32.963989	15.235457	10.526316	39.335180	40	453	51	515	102	2344	782	9013	27	83	113
41	36.494253	15.229885	11.206897	35.919540	41	500	50	635	113	2970	882	10,510	29	84	104
42	35.347432	16.918429	14.803625	32.024169	42	233	47	950	117	3468	1000	10,003	23	77	107
43	30.678466	20.353982	11.799410	34.513274	43	594	37	605	103	1769	580	9567	21	58	179
44	31.003040	18.237082	14.589666	34.650456	44	706	34	1310	108	3016	771	13,078	24	45	186
45	23.920266	17.607973	20.265781	37.209302	45	681	24	850	100	1821	491	9509	18	29	158
46	31.118881	25.524476	15.034965	25.174825	46	629	37	833	104	2782	809	9732	24	52	120
47	29.967427	16.286645	10.097720	42.671010	47	492	35	919	119	3556	874	11,449	29	55	122
48	27.102804	17.445483	11.214953	42.990654	48	647	32	368	96	1899	510	9666	23	43	138
49	20.895522	18.805970	9.552239	50.746269	49	760	30	214	99	1376	397	8228	31	43	148
50	25.196850	7.874016	9.973753	56.430446	50	558	34	363	94	2207	568	8717	32	51	111
51	20.391061	14.804469	9.497207	55.027933	51	759	26	374	65	1855	405	8160	23	38	146
52	16.257669	20.552147	8.282209	54.601227	52	687	46	763	122	3714	947	11,965	45	73	74
53	27.071823	16.574586	7.458564	48.895028	53	638	57	618	134	4775	1155	13,021	49	94	58
54	28.055556	15.555556	7.500000	48.888889	54	678	60	733	123	4535	1120	12,987	44	94	60
55	42.295082	17.377049	4.262295	35.737705	55	526	60	492	107	3403	975	13,889	52	97	45
56	41.319444	17.708333	3.819444	36.458333	56	525	60	1171	139	4009	1071	13,064	50	98	28
57	39.629630	15.185185	5.555556	38.888889	57	635	68	1736	185	5815	1411	13,318	52	112	35
58	27.065527	7.407407	3.988604	61.538462	58	233	59	1339	230	4300	1048	13,064	48	85	55
59	27.851459	13.262599	5.305040	53.050398	59	584	55	4833	2922	5617	974	46,777	45	81	49
60	20.746888	14.107884	6.224066	58.921162	60	658	62	4968	1837	5911	1257	29,916	45	85	61
61	23.624595	13.268608	8.737864	53.398058	61	679	74	2905	502	5216	1240	14,838	52	115	39
62	21.034483	16.896552	9.310345	51.724138	62	575	73	2750	242	5590	1353	13,583	52	112	38
63	18.098160	19.018405	8.588957	53.374233	63	362	69	4754	421	4987	1213	14,565	51	84	48
64	16.285714	21.428571	7.714286	54.000000	64	357	72	3806	382	5407	1235	13,869	56	103	37
65	13.714286	23.142857	6.285714	55.714286	65	402	67	3641	493	4277	1120	14,239	48	96	46
66	13.702624	22.448980	6.705539	56.268222	66	347	65	3775	451	4770	1099	15,102	42	91	44
67	28.660436	9.968847	7.476636	53.894081	67	331	66	3030	306	4857	1238	13,506	45	101	47
68	38.095238	5.714286	6.984127	48.888889	68	259	70	1900	285	5410	1477	13,843	52	115	35
69	26.689189	7.432432	8.783784	56.756757	69	511	64	3809	1005	5201	1213	19,435	48	93	55
70	18.209877	7.716049	10.185185	63.580247	70	369	73	2374	336	3656	1074	12,903	49	111	50
71	14.423077	6.089744	7.692308	71.794872	71	237	77	2590	258	5763	1443	13,132	48	125	50
72	13.750000	4.687500	7.187500	74.375000	72	339	71	2088	176	3491	986	11,579	41	116	94
73	11.075949	4.746835	14.873418	68.670886	73	594	67	1083	138	1961	629	9154	33	117	169
74	2.500000	5.000000	4.687500	4.687500	74	470	73	1805	143	3205	906	9778	36	132	183
75	6.590258	4.011461	15.472779	73.065903	75	724	64	839	99	1640	514	8444	30	99	191
76	6.532663	3.768844	13.065327	75.628141	76	607	66	1104	133	4580	329	3423	11	17	385
77	5.080214	0.000000	13.368984	81.550802	77	609	65	2031	162	4309	975	10,976	37	99	137
78	7.055961	0.000000	13.138686	79.805353	78	684	60	1655	127	3594	840	10,248	35	82	202
79	6.940063	2.839117	4.731861	85.173502	79	674	54	1460	147	1344	335	7371	19	68	257
80	20.157068	14.397906	2.094241	62.041885	80	362	79	6013	353	7347	1701	14,025	48	113	44
81	12.418301	15.032680	1.960784	69.934641	81	349	98	4418	312	5543	1353	13,047	45	159	37
82	15.335463	12.140575	3.833866	67.731629	82	500	100	6000	586	7572	1667	17,931	49	202	45

**Table 3:** Six types of ecosystems: The six ecosystems used in potential correlations in the biplots between geo- and hydrological-conditions include interdistributary, delta-plain, deltaic lake, bottomland and swamp forests, freshwater marsh, and intermediate marsh.

	Six ecosystems
1	interdistributary deltaic plain
2	St. Bernard delta-plain
3	deltaic lake
4	bottomland and swamp forests
5	freshwater marsh
6	intermediate marsh

Table 2 provides ecological and geochemical datasets used in the multivariate Principal Component Analysis (PCA). Four taxonomic groups (floodplain trees, upland trees, tidal freshwater herbs, and inland herbs) and marine (Cl, Sr, Ca) and terrestrial (Mn, K, Ti, Fe, Zn, Zr, Br) elements, developed in eighty-two rows in the data set, facilitate the testing of potential correlations between ecological and geochemical variables. Table 3 provides categorical variables for six ecosystems (interdistributary deltaic plain, St. Bernard delta-plain, deltaic lake, bottomland and swamp forests, freshwater marsh, and intermediate marsh) that are correlated with the ecological and geochemical datasets.

## 2 Experimental Design, Materials and Methods

The datasets and frames were devised to identify external forcing agest(s) driving each ecological transition and to elucidate the possibility of applying these mechanisms to current and future conditions in coastal environments, a nexus of such globally important environmental and societal stresses as coastal erosion, oceanic transgression, infrastructural vulnerability, human displacement, flooding, saline water intrusion, landward migration of coastal plants, and species extinction. Although these negative impacts are generally global, resolutions are not, as wide variability in local conditions requires site-specific solutions. In any specific location, the first step, of course, is to identify the proximate cause of the stressor, which can be driven by a variety of external factors such as eustatic sea-level rise (SLR), subsidence, climatic shifts, anthropogenic activities, extreme events, and fluvial dynamics (river course changes), all of which leave potentially identifiable ecological and geochemical imprints in the sedimentary record. This methodology has been created to identify the specific external forcing agent(s) driving environmental change at a specific site at a specific time. This is accomplished through the use of multivariate Principal Component Analysis (PCA) of sedimentary data (ecological and geochemical). The methodology involves the following steps.

Step 1. Sample collection and modern vegetation database.

The sampling site was carefully chosen after a series of reconnaissance in order to minimize sedimentary disturbance associated with anthropogenic activities (e.g., canal dredging). Plants around the study site were widely analyzed in order to establish modern pollen and vegetation databases.

Palynology-based sedimentary analyses were followed by conventional methods [9,10], creating 2821 fossil pollen datasets (Table 1). Geochemical data were analyzed using the XRF analyzer, scanning the center of the core, longitudinally at 2 cm intervals [11], creating 820 geochemical datasets (Table 2).

Step 2. Creating data frames.

In order to perform the multivariate statistical tests, pollen data (Table 1) were correlated with core-log geochemical datasets, creating eighty-two rows on an ecological and geochemical data frame (Table 2). Based on the agglomerative hierarchical procedure [7,8], six ecosystems (interdistributary, delta-plain, deltaic lake, bottomland and swamp forests, freshwater marsh, and intermediate marsh) were determined (Table 3). The "data.table()" function was employed to implement six ecosystems to core-log datasets creating eighty-two individuals.

Step 3. Multivariate PCA technique.

Multivariate statistical technique facilitated the testing of potential correlations between the marine (Cl, Sr, Ca) and terrestrial (Mn, K, Ti, Fe, Zn, Zr, Br) elements and the four taxonomic groups (floodplain trees, upland trees, tidal freshwater herbs, and inland herbs) by means of software R packages (e.g., "factoextra", "FactoMineR") [6]. The datasets in different scales were standardized as means of “scale()” function [12]. Eigenvalues measuring the amount of variation were computed with the code “get_eigenvalue()”, and visualized with the code “fviz_eig” [12]. The representation of each variable on the principal components biplots was estimated with the squared cosine function [13]. The environmental information in given geological and geochemical datasets corresponds to the total variation along the PCA scores, which were visualized graphically in biplots. An ellipse function [14] was used to plot confidence ellipses around group mean points.

Step 4. Biplot interpretation.

The biplots (SI1 to SI6) display the correlations between ecological and geochemical variables. The ellipses of categorical variables visualize the relationships between six ecosystems and hydrological conditions, with a larger ellipse indicating a larger variable [14]. The horizontal Dim1 axis represents the first principal component, while the orthogonal Dim2 axis indicates the second principal component. The PCA values elucidate the relative importance of the total variables as they reduced the dimensionality of the multivariate data elaborating unique dimensions of dataset variability. The representation of each variable in biplots indicates the importance of each variable for a given observation. The distance of a variable from the biplot center, positively correlates to relative importance.

These multivariate PCA datasets facilitate millennial-scale paleoenvironmental reconstructions, including hydrological and ecological analyses with accurate dating techniques (e.g., low-energy germanium γ-spectrometer and AMS radiocarbon [10]). The detailed information regarding hydrology, geology, vegetation, and soil chemistry over a large swath of the Louisiana coast for an extended time frame, will provide reliable insights into plant migration, relative sea-level rise, salinity variability, and climate change (SI6), thus providing significant datasets for the future wetland study.

## CRediT authorship contribution statement

**Junghyung Ryu:** Conceptualization, Data curation, Methodology, Software, Writing – original draft, Visualization. **Kam-biu Liu:** Supervision, Conceptualization, Project administration, Funding acquisition, Resources. **Terrence A. McCloskey:** Conceptualization, Validation, Writing – review & editing.

## Declaration of Competing Interest

The authors declare that they have no known competing financial interests or personal relationships that could have appeared to influence the work reported in this paper.

## Data Availability

LSWMA dataset (Original data) (Mendeley Data).
